# EDST: a decision stump based ensemble algorithm for synergistic drug combination prediction

**DOI:** 10.1186/s12859-023-05453-3

**Published:** 2023-08-29

**Authors:** Jing Chen, Lianlian Wu, Kunhong Liu, Yong Xu, Song He, Xiaochen Bo

**Affiliations:** 1 Xiamen University, Xiamen, China; 2 Tianjin University, Tianjin, China; 3 Fujian University of Technology, Fuzhou, China; 4 Institute of Health Service and Transfusion Medicine, Beijing, China

**Keywords:** Decision tree, Decision stump, Bagging, Drug combination, Interpretability

## Abstract

**Introduction:**

There are countless possibilities for drug combinations, which makes it expensive and time-consuming to rely solely on clinical trials to determine the effects of each possible drug combination. In order to screen out the most effective drug combinations more quickly, scholars began to apply machine learning to drug combination prediction. However, most of them are of low interpretability. Consequently, even though they can sometimes produce high prediction accuracy, experts in the medical and biological fields can still not fully rely on their judgments because of the lack of knowledge about the decision-making process.

**Related work:**

Decision trees and their ensemble algorithms are considered to be suitable methods for pharmaceutical applications due to their excellent performance and good interpretability. We review existing decision trees or decision tree ensemble algorithms in the medical field and point out their shortcomings.

**Method:**

This study proposes a decision stump (DS)-based solution to extract interpretable knowledge from data sets. In this method, a set of DSs is first generated to selectively form a decision tree (DST). Different from the traditional decision tree, our algorithm not only enables a partial exchange of information between base classifiers by introducing a stump exchange method but also uses a modified Gini index to evaluate stump performance so that the generation of each node is evaluated by a global view to maintain high generalization ability. Furthermore, these trees are combined to construct an ensemble of DST (EDST).

**Experiment:**

The two-drug combination data sets are collected from two cell lines with three classes (additive, antagonistic and synergistic effects) to test our method. Experimental results show that both our DST and EDST perform better than other methods. Besides, the rules generated by our methods are more compact and more accurate than other rule-based algorithms. Finally, we also analyze the extracted knowledge by the model in the field of bioinformatics.

**Conclusion:**

The novel decision tree ensemble model can effectively predict the effect of drug combination datasets and easily obtain the decision-making process.

## Introduction

In recent decades, great advances in drug development have been made with the discovery and application of new therapeutic targets [1]. However, because many diseases are complex and involve multiple target genes, a single therapy cannot cure the disease completely. Moreover, drug resistance in many cases is a major barrier to effective treatment due to the complexity of the disease. To overcome the limitations of monotherapy, combination therapy is considered a promising approach to achieving better disease control. While high-throughput screening methods can be effective in speeding up the identification of synergistic drug combinations, the increasing number of drugs every year makes it expensive and time-consuming to rely solely on experiments to determine the effect of each possible drug combination [2]. So academics have begun to use methods in the field of statistics or computers to predict the most effective combination of drugs [3].

Having a complete drug combination database is the primary condition for analyzing efficacy, so some scholars have collected various existing experimental data and summarized them into the database [4, 5]. And to better help others understand the relationship between drug resistance and drug signature attributes, they also provide visualization tools for drug combinations in the database [6–8]. With the support of databases, various technologies began to emerge. More and more machine learning techniques-based applications had been proposed from different aspects [9–12]. For example, Julkunen et al. [13] proposed comboFM, which modeled multidirectional interactions between cell lines and dose-response matrices of two drugs. Shi et al. [14] combined one-class SVM to design a two-layer multi-class classification system integrating five types of features that can discover potential drug pairs among unknown drugs. There are also some scholars use semi-supervised heterogeneous network algorithms based on graph embedding to predict the combination patterns of drugs [15]. However, these methods based on traditional machine learning often do not achieve better prediction results. To this end, some scholars have tried to give up interpretability in exchange for improved accuracy.

DeepSynergy [16] which was constructed by a feed-forward neural network with two hidden layers is one of the early deep learning algorithms used in the drug combination. MatchMaker [17] which contained three neural subnetworks was proposed by Kuru et al. Both DeepSynergy and MatchMaker have been used to predict drug synergies in recent years and proved popular. There are many similar deep learning-based algorithms used in drug prediction, and they usually have good performance [18–22]. However, the hundreds of neurons and the complex network structure make their internal logic incomprehensible. People therefore unable to conclude whether the predictions given by these models in practical applications can be trusted, which is considered very dangerous.

In order to balance the performance and interpretability of the model, scholars have made many attempts, including the ensemble algorithm based on decision trees. This algorithm not only improves the model performance by means of ensemble, but also maintains the interpretability of the decision tree. That’s why our algorithmic framework uses this approach. In addition, to avoid the problem of reduced interpretability of knowledge after integration due to excessive depth of decision trees. We replace the binary splitting of traditional decision trees with multi-branched decision stump merging.

This study proposes an interpretable method for drug combination analysis. In detail, an ensemble algorithm is designed using DS-based tree structures as base learners. Our method is applied to classifying the drug combination data and extracting interpretable rules simultaneously. The main contributions of the paper are:

- The construction of a 1956-dimensional dataset of the drug combination in two cell lines.
- An effective ensemble algorithm, which not only eliminates the class imbalance problem by balancing the sampling probability and pairwise classification scheme, but also makes the decision information more global by introducing the stump exchange strategy.
- A new tree generation algorithm based on the combination of DSs, taking the influence of the entire data set into consideration with the trees growing in shallower depth and fewer leaf nodes to maintain high interpretability.
- The knowledge extracted by the algorithm analyzed in the field of bioinformatics.

## Related work

### Application of decision trees

As a typical interpretable model, the decision tree has been successfully applied to different situations [23]. For example, Deelder et al. [24] developed a customized decision tree method called Treesist-TB, which can detect genomic variants in individual studies within aggregated datasets and model variant interactions to predict TB drug resistance. Tayefi et al. [25] extracted rules from the decision trees to maintain high accuracy and strong ability in biomarker discovery. Narayanan et al. [26] proposed a new multivariate statistical algorithm, Decision Tree-PLS (DTPLS), which improves the prediction and understanding ability of models based on local partial least squares regression (PLSR). Azagury et al. [27] developed a decision tree-based machine learning model to capture drug pairs with biological synergy as well as synergistic chemical self-assembly.

### Application of tree ensemble

The data distribution has a great impact on the decision tree structure, and the ensemble of decision trees tends to provide better stability. For example, Lu et al. [28] proposed a hybrid ensemble algorithm combining AdaBoost with a genetic algorithm for cancer gene expression data analysis. An et al. [29] developed a Network EmbeDding framework in mulTiPlex networks (NEDTP) and used it to predict novel drug-target interactions. This method first applies a random walk algorithm to the similarity network of drugs and proteins to extract and merge features in the network. Finally, drug-target interactions prediction is made using the GBDT model implemented by LightGBM using drug and protein signatures. Xuan et al. [30] proposed a new gradient boosting decision tree-based method named DTIGBDT and used it to predict drug candidate-target interactions. The algorithm divides the path between the drug and target into multiple classes through the topological information of the drug-target heterogeneous network and constructs a model based on gradient a boosting decision tree. Ma et al. [31] combined a random forest algorithm and Shapley Additive exPlanation to predict the response of hepatocellular carcinoma under combination therapy. Hadi et al. [32] investigated the combination of quantitative computed tomography parametric imaging with the AdaBoost decision tree to predict how LABC tumors respond to NAC.

However, some of these algorithms take lots of time because of their complexity, and some do not achieve ideal results in pursuit of interpretability. So we propose an algorithm with faster running speed and better interpretability under the premise of ensuring good performance.

## Method

**Fig. 1:**
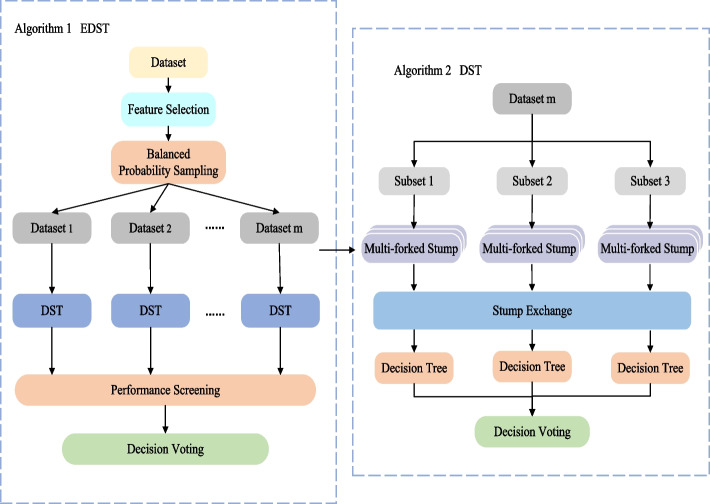
Overall flow chart of EDST algorithm

This section gives the details about the proposed adaptive ensemble method, as shown in Fig. 1. The right side of the figure shows the process of dividing the subdatasets through the one-vs-one method and training their decision trees for final voting. On the left is the process of data preprocessing and how to integrate the decision-making results of each subdatasets. The specific steps are as follows. Assume $D=\{(x_{p1},x_{p2},\ldots ,x_{pv}, y_p)\}_{p=1}^n$ represents the data set containing *v* features and *n* samples, where $y_p$ denotes the label of the *p*-th sample. Algorithm 1 describes the specific contents of the entire ensemble algorithm. In order to reduce redundancy and speed up operation, our algorithm first selects the features of the dataset in step 1. Unlike simple random sampling in traditional random forests, our algorithm generates sub-datasets by the following sampling probability $\alpha$:

1 $$\begin{aligned} \alpha _j = \frac{\frac{1}{N_j}}{\sum _{j=1}^C\frac{1}{N_j}}, \end{aligned}$$

where $N_j$ represents the number of samples of the *j*-th class in dataset *D* and *C* represents the number of classes in dataset *D*. By assigning different sampling probabilities to samples of different classes, the samples in minority classes get higher sampling probability, so as to overcome the class imbalance problem. In this way, $2\times m$ replicates give $2\times m$ different subsets of samples. Then, a stump-based decision tree (DST) is trained on each subset of samples and the top *m* trees that work best on the training set *D* are retained. Finally, the results of these *m* trees probabilistically vote to obtain the final output.

**Fig. 2:**
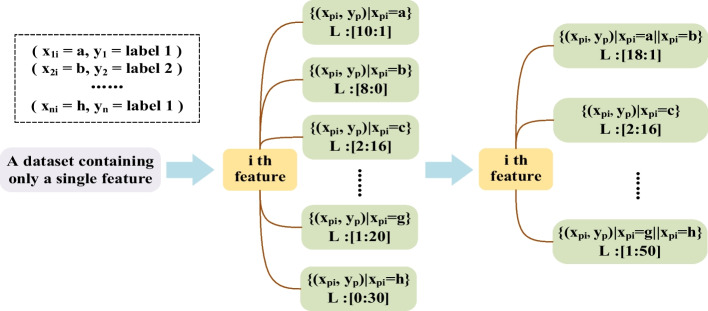
An example of the tree stump generation

After introducing the overall framework of the integration algorithm, the specific process of generating a complete decision tree (DST) will be followed (steps 4–24). To increase accuracy for multi-classification problems, our algorithm first divides the dataset into several sub-datasets by sampling in the one-vs-one scheme, which pairs up *c* classes to generate $c(c-1)/2$ binary classification tasks (steps 4–5). Then, the algorithm needs to generate a corresponding stump for each feature (step 6). Fig. 2 shows the general process of generating a DS on $f_i$, where $\{(x_{pi},y_p)|x_{pi}=a\}$ represents a set of samples taking value *a* and *L* represents the proportion of relevant labels in the group. For the continuous feature $f_i=\{x_{1i},x_{2i},\ldots ,x_{ni} \}^T$ which denotes the *i*-th feature vector, all training samples are sorted by their values in $f_i$, forming a set of intervals. Samples are divided into different intervals from smallest to largest according to their distribution. The label that appears most often in each interval is set as the final label. After that, the labels of all the intervals are checked, and the adjacent intervals with the same label are combined to form a group. For example, the top green square contains all samples with the *i*-th feature value *a*, where label 1 and label 2 are 10 to 1. Then, by comparing the proportion of samples in the adjacent green squares, the algorithm determines that the samples contained in the $x_{pi}=a$ and $x_{pi}=b$ squares are of the same category, and can be combined.

Next, we draw on ideas from the Genetic Algorithm (GA) to cross-mutate stumps in different subsets of samples (step 7). Based on the classification results of the stumps, the similarity of the two stumps is calculated by Eq 2. The 50% stump with the highest sum of similarities is screened out, which may have less information. Since there are a total of $2\times m$ subsets of data, $2\times m$ sets containing the highest similarity stumps can be obtained. If the stump generated by the same feature appears in multiple sets at the same time, the algorithm randomly exchanges them so that information can be passed between different subsets of data.

2 $$\begin{aligned} {\textrm{Similarity}} = \frac{\sum _{p=1}^n(x_p\times y_p)}{\sqrt{\sum _{p=1}^n(x_p)^2 \times \sum _{p=1}^n(y_p)^2}}, \end{aligned}$$

After the stump exchange is completed, the algorithm evaluates the classification performance of their stumps and selects the stump with the best performance as the root node stump (steps 8–13). Here use the Gini index as the evaluation method for each node of the DS-based decision tree, and its formula is as follows:

3 $$\begin{aligned} {\textrm{Gini}} = \sum _{z}^u(1-\sum _{j}^cp_{j,z}^2), \end{aligned}$$

where *c* is the number of classes in the present sub-dataset, *u* is the number of leaves of the feature tree stump, and $p_(j,z)$ represents the ratio of the number of samples in the *j*-th class to the total number of samples in the *z*-th leaf node. This Gini index is modified by removing the penalty on the number of samples in the leaf to split the samples better. Finally, the stump is spliced in a loop iteration to generate an entire decision tree (steps 14–21) until the sample contained in a branch is smaller than $\theta$, which is set to *n*/10 in our algorithm.

In our algorithm, the use of DSs as the elements to build a decision tree makes the tree gain global information instead of local information on the data at each split, to enhance the tree’s generalization ability. The results of each tree are fused by the majority soft voting strategy to get the final decision. In this way, the base classifier DST required for the ensemble is obtained.
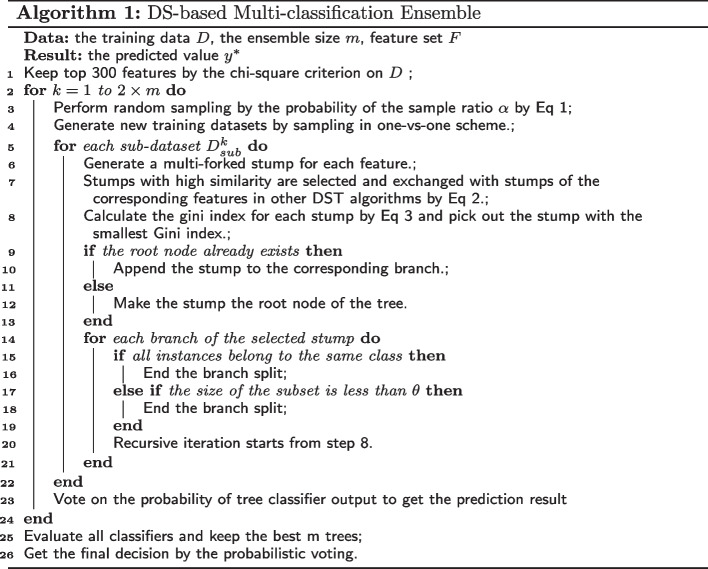


## Experiment

### Datasets

In the dataset, a sample represent a drug combination on a particular cancer cell line, i.e., a drug combination-cell line pair. The samples are collected from DrugComb database (v1.4) [4]. To predict cell line-specific synergistic drug combinations, samples from different cell lines are modeled separately. For a sample, DrugComb contains four types of synergy scores, including Bliss Independence, Highest Single Agent, Loewe Additivity, and Zero Interaction Potency, where positive values represent synergy and negative values represent the antagonism of the drug combination. In this study, samples are divided into three categories (Synergy, Antagonism, and Additive) based on all mentioned four synergy scores. Synergy or Antagonism represent those samples whose four synergy scores are both positive or negative numbers, respectively, while the remaining samples are classified as Additive. Then, the samples with missing features are removed. Finally, two typical cell lines, HT29 colorectal cell line and A375 melanoma cell line, that have the largest sample capacity are selected to construct the data set. Table 1 lists the number of samples in each class and each cell line. It can be observed that the number of samples in the Additive class is greater than the sum of the samples of the other two classes. This problem will affect model training and prediction.

**Table 1:** Data distribution in each cell line

Name	Combination medication dataset			
	Dataset size	Antagonism	Additive	Synergy
HT29	(725, 1956)	134	451	140
A375	(130, 1956)	21	74	35

The feature data are cell line-specific drug-inducible gene expression data extracted from L1000 project of the LINCS database. LINCS L1000 is the expanded CMap (Connectivity Map) that can be used to discover mechanism of action of small molecules, functionally annotate genetic variants of disease genes, and inform clinical trials [33]. In 2006, Lamb et al. piloted the CMap concept by treating cells with 164 drugs and tool compounds, and then performing mRNA expression profiling using Affymetrix microarrays [34]. However, the small scale of CMap limited its utility [33]. Therefore, the CMap team proposed a new approach, L1000, to obtain gene expression profiling based on a reduced representation of the transcriptome. L1000 is a low-cost, high-throughput method that only needs 1,058 probes for 978 landmark transcripts and 80 control transcripts, making it well-suited for a large-scale Connectivity Map. The first release of 1,319,138 L1000 profiles are termed CMap-L1000v1, which serves as the data source for our study. In this study, the cell line-specific drug-inducible gene expression data of 978 landmark genes from the LINCS L1000 database are used to construct the feature dataset. The 978 landmarks have been shown to be sufficient to recover 80% of the information in the full transcriptome by Subramanian et al. We first obtain the Level 5 data from LINCS L1000 project, which contain the z-scores of gene expressions with multiple doses and times. For the data of different doses and times of the same drug in Level 5, we get the unique gene expression by applying the moderated z-score approach, which is used to derive the consensus replicate signatures from Level 4 in LINCS L1000 project [33]. More specifically, the z-scores are weighted and averaged according to Spearman correlations. Finally, to obtain the feature of a sample, we splice together the 978-dimensional gene expression profiles of two drugs in combination, resulting in a 1956-dimensional feature vector. Excessive dimensionality of features is also a common problem in drug combination datasets [9]. This may increase the complexity of data processing and affect the prediction performance of the model.

The above-mentioned two major problems of unbalanced dataset samples and too high feature dimensions can be well solved in our algorithm through one-vs-one classification, probability sampling, and feature selection.

### Classification performance

In this section, we verify the classification effect of our method with the above-mentioned dataset and compare it with other common algorithms and some tree-based methods, including decision tree, XGboost, traditional random forest, SVM, KNN, MLP, etc. The neural network model that is difficult to explain is not the focus of our attention. So we only choose Deep Synergy and MatchMaker, which are widely used for drug synergy prediction among the classifiers based on deep learning. In addition, in order to facilitate comparison, we also built a simple single-layer network structure with only the minimum parameter configuration. These machine learning methods are implemented through the scikit-learn. When the two deep learning methods of Deep Synergy and MatchMaker are used, we do not modify the default parameters except for changing the activation function from linear to softmax. To ensure fairness, we use the same sample set for all methods. We used 80% of the samples in the combination drug data of the two cell lines as the training set and the remaining 20% as the test set and adopted a five-fold cross-validation method. The specific files of the dataset and algorithm implementation can be found at https://github.com/chenjing13/EDST.

Since the dataset contains a large number of addition effect samples, which are often the ones that we do not need to pay too much attention to, we focus on the performance evaluation indicators such as AUC, F1 score, and the minority class metrics of recall and precision, which are also calculated through metrics function in the scikit-learn library, the formula is as follows:

4 $$\begin{aligned} {\textrm{Precision}}&= \frac{\textrm{TP}}{\textrm{TP}+\textrm{FP}}, \end{aligned}$$

5 $$\begin{aligned} {\textrm{Recall}}&= \frac{\textrm{TP}}{\textrm{TP}+\textrm{FN}}, \end{aligned}$$

6 $$\begin{aligned} {\textrm{F1 score}}&= \frac{2\cdot \textrm{Precision}\cdot \textrm{Recall}}{\textrm{Precision}+\textrm{Recall}}, \end{aligned}$$

7 $$\begin{aligned} {\textrm{AUC}} &= \frac{\sum _{i\in k}^crank_i-\frac{(\textrm{TP}+\textrm{FP})(\textrm{TP}+\textrm{FP}+1)}{2}}{(\textrm{TP}+\textrm{FP})(\textrm{TN}+\textrm{FN})}, \end{aligned}$$

where TP/FP indicates that the positive predictions are true/false, and TN/FN indicates that the negative predictions are true/false. $rank_i$ indicates the position of the *i*-th sample sorted by probability from smallest to largest. These indicators are used because all of the above datasets are unbalanced, and the more important synergies and antagonisms account for a small number of samples in the total sample size.

Tables 2 and 3 show the results obtained from different cell lines. The F1 score* is calculated by the algorithm on the recall and precision metrics in the Antagonism and Synergy categories. For the classification results of different categories, the EDST model proposed in this paper outperforms other methods on most metrics, especially on AUC and F1 score. Furthermore, our method yields higher recall for both antagonism and synergy samples. Although performance on precision is not always the optimal, precision and recall have some degree of conflict, especially with unbalanced datasets. From the table, we can see that SVM, KNN, and so on all get a recall of the additive higher than 0.8. That is because they classify more samples as additive, which results in fewer minority classes being identified. It can partly explain why these algorithms may show slightly higher in the precision of minority classes than ours. So AUC and F1 score can be the good criterion in this case. Finally, to observe the effect of each algorithm more clearly, we rank it, where Rank represents the ranking of each algorithm on AUC, F1 score, and all classes of recall and precision, and Rank* indicates the ranking of each algorithm on AUC, F1 score*, and the recall and precision of the class with fewer samples. he smaller the Rank or Rank* of an algorithm, the better it is. We can see that whether on Rank or Rank*, our algorithm is ahead of others. All the hyperparameters used in the model are shown in Table 4.

While our method does not always perform better on summed samples, it can achieve better performance in the minority class. That is, our method slightly sacrifices the performance of the majority class to guarantee results for the minority class. The method achieves the highest AUC and F1 score, which further confirms that the method can handle the class imbalance problem well.

**Table 2:** Classification results of algorithms on the colorectal cell line dataset HT29

HT29	AUC	F1 score	F1 score*	Recall			Precision			Rank	Rank*
				Additive	Antagonism	Synergy	Additive	Antagonism	Synergy		
SVM	0.6363	0.4294	0.2879	0.8247	0.1578	0.2929	0.6462	0.2933	0.5040	5.1	5.6
KNN	0.5769	0.3892	0.2188	0.8714	0.0969	0.2143	0.6464	0.3128	0.4242	6.2	7.6
XGB	0.6523	0.4494	0.3143	0.8224	0.1860	0.3143	0.6373	0.3279	**0.5178**	4.0	3.3
MLP	0.6441	0.3961	0.2411	0.8602	0.1123	0.2214	0.6347	0.3694	0.4995	5.3	5.6
DT	0.5673	0.4018	0.2922	0.6251	0.2906	0.2929	0.6285	0.2746	0.3110	7.0	6.3
GDBT	0.6574	0.4257	0.2802	0.8115	0.1712	0.2786	0.6495	0.3091	0.4344	4.8	5.3
RF	0.6569	0.4041	0.2468	0.8449	0.2017	0.1571	0.6404	**0.3957**	0.3953	4.8	5.3
Single-layer Network	0.4829	0.2101	0.1821	0.2985	0.4966	0.1643	0.5861	0.1602	0.0913	9.5	9.1
DeepSynergy	0.5073	0.3739	0.0411	**0.9665**	0.0200	0.0240	0.6203	0.3400	0.3000	7.8	9.1
MatchMaker	0.6142	0.2922	0.3040	0.2067	**0.6496**	0.4286	0.4965	0.2434	0.1801	7.7	5.5
EDST	**0.6593**	**0.4603**	**0.42825**	0.4189	0.5148	**0.6286**	**0.7231**	0.3223	0.3824	**3.3**	**2.8**

**Table 3:** Classification results of algorithms on the melanoma cell line dataset A375

A375	AUC	F1 score	F1 score*	Recall			Precision			Rank	Rank*
				Additive	Antagonism	Synergy	Additive	Antagonism	Synergy		
SVM	0.5256	0.3291	0.2164	0.5800	0.2500	0.1714	0.5463	0.2500	0.1950	8.3	9.0
KNN	0.5729	0.3934	0.2678	0.7867	0.1400	0.2286	0.5787	**0.5000**	**0.4800**	4.7	5.5
XGB	0.5946	0.3905	0.3104	0.5543	0.3000	0.3714	0.5745	0.2500	0.3274	5.6	4.6
MLP	0.6441	0.3980	0.2921	0.6771	0.3000	0.2286	0.5828	0.2733	0.3800	4.2	4.8
DT	0.5673	0.3330	0.2679	0.4305	0.3429	0.2500	0.5152	0.2756	0.2133	7.0	5.6
GDBT	0.6574	0.3538	0.2620	0.5552	0.2857	0.2500	0.5635	0.2530	0.2600	6.1	5.8
RF	0.6569	0.4000	0.3070	0.6762	0.2000	0.3143	0.5789	0.3300	0.4321	3.8	4.0
Single-layer Network	0.5086	0.2242	0.0994	0.6000	0.2857	0.0500	0.4325	0.1288	0.0125	9.2	10.0
DeepSynergy	0.5071	0.3791	0.2345	**0.8124**	0.1500	0.2286	0.6037	0.3000	0.3167	5.8	7.8
MatchMaker	0.5120	0.2124	0.2346	0.3000	0.4800	0.1500	0.1435	0.3308	0.0429	8.3	7.0
EDST	**0.6625**	**0.4680**	**0.4220**	0.5286	**0.5143**	**0.3900**	**0.6216**	0.4593	0.3319	**2.5**	**1.6**

**Table 4:** Model Hyperparameters

Number of trees	100
Proportion of the exchange of stumps	10%
Maximum depth of the decision tree	10
Minimum number of samples for leaf nodes	10%

### Performance of DS-based tree

**Fig. 3:**
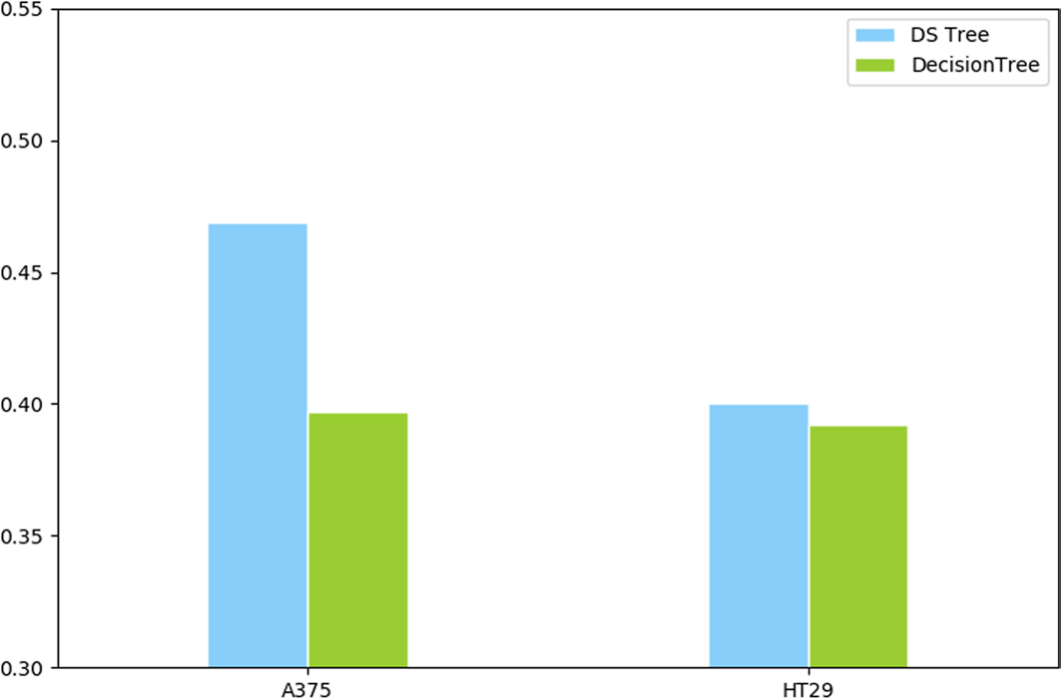
DS-based tree and traditional decision tree F1 score comparison

Again, we are using the F1 score as a judging metric to compare the performance differences between the DS-based decision tree and the traditional one without an ensemble. The results are shown in Fig. 3. The F1 score of the DS-based tree is always better than that of the traditional decision tree on the dataset of both cell lines. This means that our algorithm performs better than traditional decision trees on the balance of predictions between the two cell line samples.

### Ablation experiment

#### Advantages of the one-vs-one scheme

Table 5 gives the classification results of DST with and without the one-vs-one scheme. Whether it is in the F1 score or the recall and precision of minority classes, those that have used the one-vs-one scheme have better results than those that have not used the one-vs-one scheme, which indicates that some samples not identified by the feature stump can be partially correctly identified after using the one-vs-one scheme.

**Table 5:** Dst module with and without using the one-vs-one scheme on HT29 and A375

HT29	F1 score	Minority class recall		Minority class precision	
With one-vs-one	0.4598	0.3872	0.4857	0.3301	0.3429
Without one-vs-one	0.4539	0.3202	0.4286	0.3076	0.3938

A375	F1 score	Minority class recall		Minority class precision	
With one-vs-one	0.3947	0.3429	0.3500	0.4278	0.2086
Without one-vs-one	0.3747	0.2571	0.3000	0.3217	0.3357

#### Advantages of the stump exchange module

Table 6 shows the performance comparison of the algorithm EDST with or without using the stump exchange module. We can see from the table that the EDST algorithm using the stump exchange module performs significantly better on the F1 score and the recall and precision indicators of one minority class, but does not significantly improve on the other minority class. We suspect that this may be due to the data of the Synergy class being insensitive to distribution.

**Table 6:** EDST algorithm with and without using the stump exchange module on HT29 and A375

HT29	F1 score	Minority class recall		Minority class precision	
With stump exchange	0.4603	0.5148	0.6286	0.3223	0.3824
Without stump exchange	0.4470	0.4926	0.6214	0.2917	0.3694

A375	F1 score	Minority class recall		Minority class precision	
With stump exchange	0.4680	0.5143	0.3900	0.4593	0.3319
Without stump exchange	0.4470	0.4286	0.3900	0.3979	0.3652

### Determination of feature selection number

**Fig. 4:**
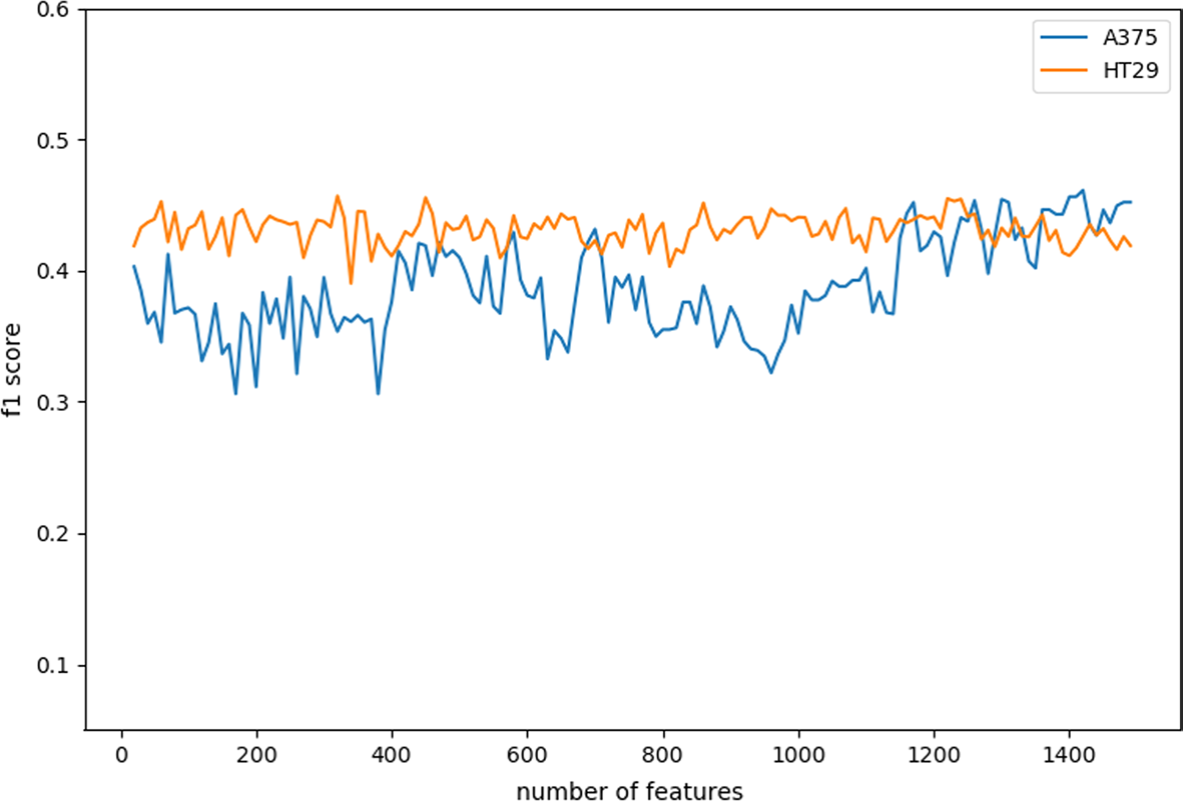
The relationship between the number of features selected and the F1 score in the EDST algorithm

**Fig. 5:**
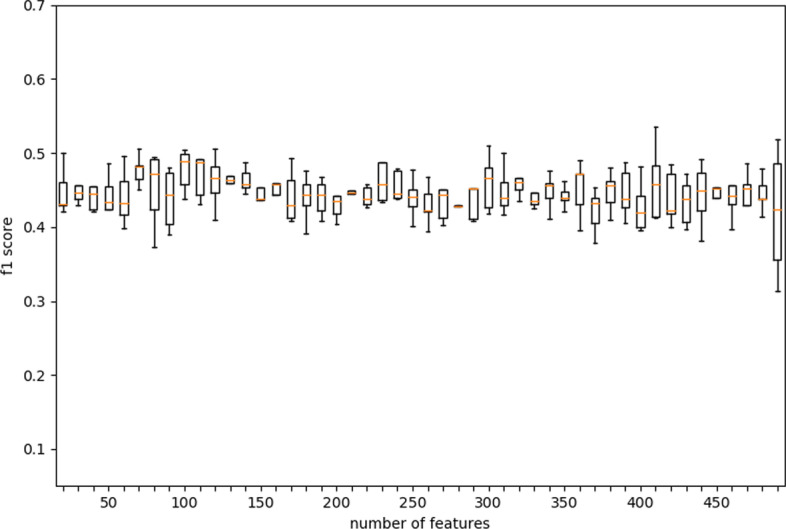
The relationship between the number of features selected and the variance in the EDST algorithm on HT29

**Fig. 6:**
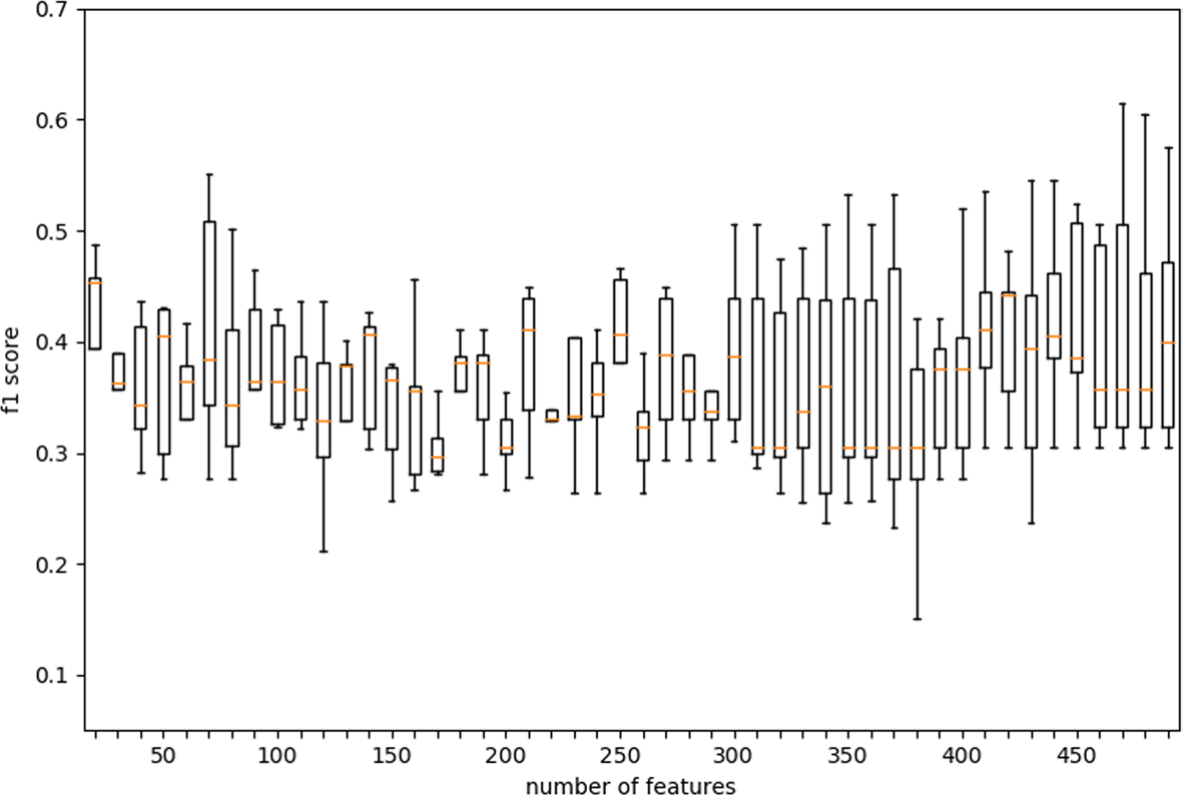
The relationship between the number of features selected and the variance in the EDST algorithm on A375

To solve the high-dimensional problem of the data and reduce the time and storage costs of the algorithm, we added a feature selection module and found the optimal number of features through experiments. We used chi-square as the criterion for feature selection and used five-fold cross-validation to look at the relationship between the number of selected features and the F1 score, respectively. In the experiment, we first worked out the correlation between the F1 score and the number of selected features and found that increasing the number of features did not significantly improve the performance of the algorithm (Fig. 4). This shows that our algorithm can perform well using only very few features. On the other hand, more features would greatly reduce the efficiency of the algorithm. Then we found by ANOVA that when the number of features is set to around 300, the variance of the algorithm is small (Figs. 5, 6). We believe that too many or too few features may increase or decrease the selection range of tree node features, thereby increasing the instability of the tree. Therefore, 300 is finally determined as the number of features selected by the algorithm.

### Evaluation of interpretability

To compare the interpretability of two trees with different growth patterns, we calculated the average maximum depth and number of leaf nodes for the two trees under five-fold cross-validation, respectively. The depth of the tree is used to represent the number of features used in a single decision, and a shallower tree means a shorter length of the generated rules. The number of leaf nodes can represent the complexity of the model to a certain extent. As can be seen from Table 7, the average maximum depth and number of leaf nodes of trees grown using our method are half of those of traditional decision trees. This means that DS-Based Trees have better interpretability than traditional decision trees. Next, we extract the rules of each tree according to the decision path and calculate the accuracy and coverage of each rule using the following formulas:

8 $$\begin{aligned} {\textrm{score}} = \frac{2*R_{coverage}*R _{accuracy}}{R_{coverage}+R_{accuracy}}, \end{aligned}$$

where $R_{coverage}$ and $R_{accuracy}$ represent the coverage and the accuracy of the rule R. The extracted rules are shown as *“IF 0.59* < *feature 1695* $\le$ *0.66 AND 0.52* < *feature 431* $\le$ *1.00 THEN PREDICT Additive THAN Synergy; IF feature 1604* $\le$ *0.37 AND feature 404* $\le$ *0.71 THEN PREDICT Additive THAN Antagonism”*, which means features 1695 and 431 distinguish the sample as the additive instead of the synergy and features 1604 and 404 distinguish it as the additive instead of the antagonism. Finally, we rank all the rules using Eq. 8 and select the top to analyze their biological significance.

**Table 7:** Size comparison of the two trees

Name	Maximum depth of tree		Number of leaf nodes	
	DS-based tree	Decision tree	DS-based tree	Decision tree
HT29	4.4	16	78.6	185.6
A375	3.6	9.8	31.6	46.6

For example, in A375 melanoma cell line, the most important rule extracted for classifying synergy and other classes is *“IF 0.2088* < *feature 1237* $\le$ *0.3578 AND 0.3618* < *feature 142* $\le$ *0.6430 THEN PREDICT Synergy THAN Additive; IF 1.0* < *feature 1850 THEN PREDICT Synergy THAN Antagonism”*. It is observed that the differential expression of features 1237, 142, and 1850 is crucial in the A375 cell line. The features 1237, 142 and 1850 represent the expression value of gene KIT, STX1A, and UFM1, respectively. Among them, genes KIT functions in the regulation processes of cell proliferation, migration, stem cell maintenance, differentiation and the occurrence of melanoma and some other cancers [35]. It is reported that KIT might be an important tumor-promoting factor that associated with metastasis and overall poor prognosis in the A375 cell line [36]. The differential expression rule of gene KIT extracted by EDST might be the key factor in the study of the A375 melanoma cell line. In HT29 colorectal cancer (CRC) cell line, the rule of *“IF 0.6469* < *feature 1305* $\le$ *0.7266 THEN PREDICT Synergy THAN Additive; IF 0.7260* < *feature 1225* $\le$ *1.0 THEN PREDICT Synergy THAN Antagonism”* is extracted as an important rule to the synergy prediction process. The features 1305 and 1225 represent the expression value of genes DDR1 and NFKB2. DDR1 has been shown to be highly expressed in most colon adenocarcinomas and appears as an indicator of worse event-free survival [37]. Arfi et al. suggested that the frequent high expression of DDR1 in colon cancer can be explored as a potential therapeutic target in this indication [37].

### Gene ontology (GO) biological processes and KEGG pathway enrichment

**Fig. 7:**
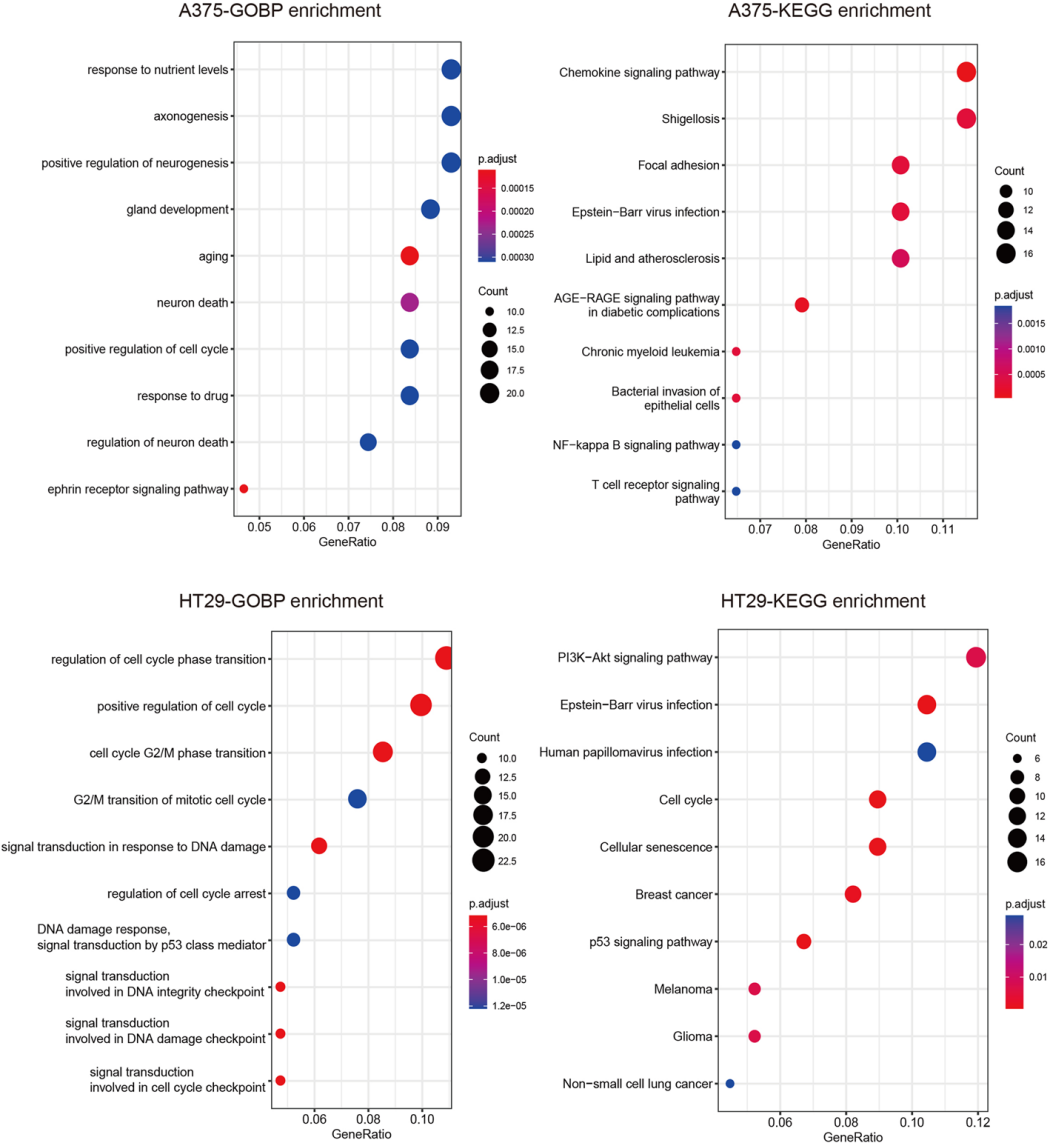
Gene Ontology (GO) biological processes and KEGG pathway enrichment on HT29 and A375

To explore the influence of drug-induced gene expression on synergy, the key features affecting the prediction process are selected, and the genes involved in the key feature subsets are further investigated in this subsection. The importance of each feature for prediction is determined by the selected frequency in the EDST. Based on the contribution value, the features ranked among the top 217 or 223 are selected on A375 or HT29 cell line respectively. The extracted features make about 90% contribution to the two cell lines. To summarize the characteristics of genes involved in these contributing features, the Gene Ontology biological processes and KEGG pathway enrichment among these features are investigated. The top 10 enrichment results are shown in Fig. 7 (adjusted P-value $< 0.05$). On the A375 cell line, the most enriched biological processes are response to nutrient levels, axonogenesis and positive regulation of neurogenesis. The most enriched pathways are the Chemokine signaling pathway, Shigellosis and Focal adhesion. But on the HT29 cell line, the enrichment results are different. The most enriched biological processes are regulation of cell cycle phase transition, positive regulation of cell cycle and cell cycle G2/M phase transition. The most enriched pathways are the PI3K-Akt signaling pathway, Epstein-Barr virus infection and Human papillomavirus.

### Leave drug combinations out

The leave drug combinations out method is a common cross-validation strategy in the field of bioinformatics [16]. In this method, the dataset is first divided into t groups according to drug types. Then, when dividing the training data and test data for each group, all samples containing the drugs in the group are used as the test data, and the others are used as the training data. In this way, we can evaluate the performance of the model in the presence of unknown drugs. In this experiment, we set t to 6. The results of our algorithm are compared with those of other algorithms, which are shown in Tables 8 and 9. From these tables, we can see that our algorithm is comparable to or better than other algorithms in the AUC and F1 score and also ahead of other algorithms in the ranking of multiple indicators. This experiment illustrates from another perspective how our algorithm performance is due to other algorithms.

**Table 8:** Classification results of algorithms using leave drug combinations out on HT29

HT29	AUC	F1 score	F1 score*	Recall			Precision			Rank	Rank*
				Additive	Antagonism	Synergy	Additive	Antagonism	Synergy		
SVM	0.5963	0.3566	0.1974	0.8117	0.1560	0.1381	0.6231	0.2758	0.3253	5.8	5.8
KNN	0.5574	0.3429	0.1641	0.8660	0.1154	0.1147	0.6306	0.3209	0.2517	6.3	8.0
XGB	0.6046	0.3559	0.1864	**0.8737**	0.1145	0.1378	0.6258	0.3090	**0.4061**	4.7	5.8
MLP	0.5989	0.3448	0.1826	0.8462	0.1190	0.1418	0.6220	0.3028	0.3072	6.1	6.3
DT	0.5067	0.3710	0.2527	0.6153	0.2574	0.2611	0.6238	0.2332	0.2597	6.1	6.0
GDBT	0.6073	0.3655	0.2009	0.8641	0.1424	0.1435	0.6374	0.3020	0.3738	3.7	4.5
RF	0.6121	0.3451	0.1884	0.8180	0.1137	0.1763	0.6279	0.2123	0.3260	5.7	6.1
Single-layer Network	0.5284	0.2031	0.2482	0.3675	0.1728	**0.4745**	0.3439	0.1454	0.2572	8.1	6.0
DeepSynergy	0.5118	0.2526	0.0576	0.8470	0.1346	0.0000	0.6155	0.1007	0.0000	9.1	10.1
MatchMaker	0.5777	0.3124	0.3177	0.3759	0.5420	0.3039	0.5733	0.2605	0.2483	7.1	5.0
EDST	**0.6129**	**0.4159**	**0.3283**	0.5945	**0.3464**	0.3453	**0.6499**	**0.3504**	0.2746	**2.8**	**2.1**

The symbol [bold] indicates the highest value in the same evaluation indicator

**Table 9:** Classification results of algorithms using leave drug combinations out on A375

A375	AUC	F1 score	F1 score*	Recall			Precision			Rank	Rank*
				Additive	Antagonism	Synergy	Additive	Antagonism	Synergy		
SVM	0.5128	0.2565	0.1858	0.5351	0.2106	0.1655	0.5014	0.1139	0.2535	9.0	8.5
KNN	0.5487	0.2940	0.1568	0.6722	0.2153	0.0625	0.5445	0.2354	0.1250	8.1	8.6
XGB	0.6226	0.3924	0.2792	0.7867	0.3134	0.2060	0.6089	0.4181	0.1857	4.0	4.3
MLP	0.6039	0.3989	0.3320	0.7193	0.3244	0.2286	0.6109	**0.4274**	0.4037	3.0	2.6
DT	0.5433	0.3813	0.3145	0.5745	0.2805	**0.3667**	0.5600	0.2718	0.3400	6.0	5.0
GDBT	0.6141	0.4037	0.3114	0.7990	0.2887	0.1810	0.5809	0.5357	0.3889	3.5	3.6
RF	0.5820	0.3629	0.2521	0.8190	0.2203	0.1298	0.5694	0.3258	**0.5750**	5.3	5.6
Single-layer Network	0.5077	0.2221	0.1408	0.4365	0.2619	0.1667	0.3684	0.1580	0.0519	9.6	9.0
DeepSynergy	0.5097	0.2906	0.0791	**0.9627**	0.0000	0.1005	0.5703	0.0000	0.3730	7.7	9.6
MatchMaker	0.5728	0.2515	0.1778	0.4577	**0.3839**	0.1944	0.5980	0.1469	0.1099	6.8	6.5
EDST	**0.6278**	**0.4236**	**0.3539**	0.6540	0.3330	0.3190	**0.6113**	0.4009	0.3732	**2.7**	**2.3**

The symbol [bold] indicates the highest value in the same evaluation indicator

## Conclusion

In this paper, we collected and constructed a combined drug dataset of colorectal cell lines and melanoma cell lines and proposed a novel stump-based decision tree ensemble algorithm for synergistic drug combination prediction. Extensive experiments showed that the decision tree generated by our algorithm is more interpretable than the traditional decision trees, and the use of ensembles can effectively improve the identification accuracy of minority classes in drug combinations and reduce the interference of large classes on samples. Finally, we showed the analysis results of the algorithm in the field of bioinformatics.

## Data Availability

Specific datasets and algorithm implementations can be found at https://github.com/chenjing13/EDST.
